# The influence of regional basic science campuses on medical students' choice of specialty and practice location: a historical cohort study

**DOI:** 10.1186/1472-6920-9-29

**Published:** 2009-06-06

**Authors:** James J Brokaw, Christina A Mandzuk, Michael E Wade, Dennis W Deal, Mary T Johnson, Gary W White, Jeffrey S Wilson, Terrell W Zollinger

**Affiliations:** 1 Office of Medical Student Affairs, Indiana University School of Medicine, 635 Barnhill Drive, MS-164, Indianapolis, Indiana 46202, USA; 2 The EMMES Corporation, Rockville, Maryland, USA; 3 Indiana State Department of Health, Indianapolis, Indiana, USA; 4 Department of Microbiology and Immunology, Indiana University School of Medicine, Terre Haute, Indiana, USA; 5 Department of Anatomy and Cell Biology, Indiana University School of Medicine, Evansville, Indiana, USA; 6 Department of Geography, Indiana University-Purdue University, Indianapolis, Indiana USA; 7 Bowen Research Center, Indiana University School of Medicine, Indianapolis, Indiana, USA

## Abstract

**Background:**

Indiana University School of Medicine (IUSM) employs eight regional basic science campuses, where half of the students complete their first two years of medical school. The other half complete all four years at the main campus in Indianapolis. The authors tested the hypothesis that training at regional campuses influences IUSM students to pursue primary care careers near the regional campuses they attended.

**Methods:**

Medical school records for 2,487 graduates (classes of 1988–1997) were matched to the 2003 American Medical Association Physician Masterfile to identify the medical specialty and practice location of each graduate. Multivariate logistic regression was performed to assess the effect of regional campus attendance on students' choice of medical specialty and practice location, while simultaneously adjusting for several covariates thought to affect these career outcomes.

**Results:**

Compared to Indianapolis students, those who attended a regional campus were somewhat more likely to be white, have parents with middle class occupations, and score slightly lower on the Medical College Admission Test. Any such differences were adjusted for in the regression models, which predicted that four of the regional campuses were significantly more likely than Indianapolis to produce family practitioners, and that five of the regional campuses were significantly more likely than the others to have former students practicing in the region. When analyzed collectively, attendance at any regional campus was a significant predictor of a primary care practice located outside the Indianapolis metropolitan area.

**Conclusion:**

Attending a regional campus for preclinical training appears to increase the likelihood of practicing primary care medicine in local communities.

## Background

The proportion of U.S. medical graduates matching into primary care residency programs–internal medicine, family medicine, and pediatrics–has fallen sharply since 1998 [1]. This trend, coupled with anticipated shortages in the physician workforce [2], have renewed worries among policy-makers of a looming health care crisis resulting from inadequate access to primary care medicine [3]. Of particular concern are the medically underserved rural and inner-city areas, which are likely to suffer even greater shortages of primary care physicians if this negative trend continues [4].

How physicians-in-training choose their medical specialties and how they decide where to practice medicine are not merely of academic interest. As the nation struggles with ever-greater health care costs, an aging "boomer" generation, and changing ethnic demographics, understanding the factors that determine the composition and distribution of the physician workforce becomes increasingly relevant to the national health care agenda. Concerned that a physician shortage will develop over the next few decades, the Association of American Medical Colleges has called for a 30% increase in medical graduates by 2015 [5]. But simply boosting physician supply will not correct the growing imbalance of primary care practitioners nor the current mal-distribution of physicians. It is more important to know how to get the right mix of physicians to practice where they are most needed. To accomplish this goal will require a better understanding of the complex interplay of variables that influence medical students' career choices.

Several factors have been proposed to explain the declining interest in primary care, such as lower remuneration for services provided by primary care physicians compared to sub-specialists, increased educational debt for medical students, and the demands of primary care practice [6]. Researchers have found that a perception of "controllable lifestyle"–defined as time for leisure, family, or avocational purposes–accounted for most of the variability in the specialty choice of recent U.S. medical school graduates [7]. Thus, the long and irregular hours necessitated by primary care practice are viewed by many students as a major deterrent in selecting a primary care field. Nevertheless, some students do choose careers in primary care medicine and do choose to practice in medically underserved areas. The influences that favor such choices can be attributed to personal characteristics of the students [8,9] as well as to educational programs designed to foster interest in primary care practice in rural or inner-city locations [10,11].

**Figure 1:**
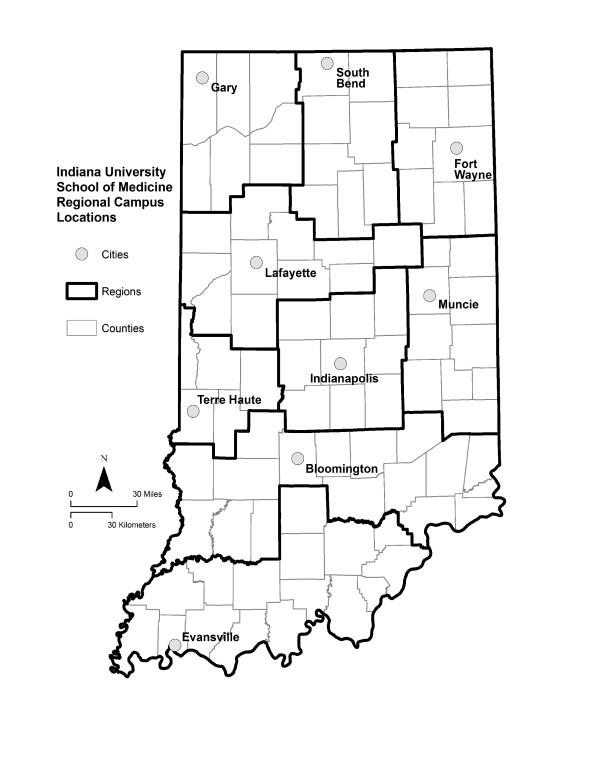
**The Indiana University statewide system of medical education**. There are eight regional basic science campuses located outside the main campus at Indianapolis, where all students receive their clinical training. Half of the matriculating students complete their first two years of medical school at one of the regional campuses, and then transfer to Indianapolis for their final two years. The other half completes all four years at Indianapolis. The dark lines denote the campus regions used in this study. The socio-demographic characteristics of the counties comprising these regions can be found at: .

Another possible influence on career choice is training at regional campuses, away from the major medical centers [12]. The Indiana University School of Medicine (IUSM) responded to the perceived physician shortage of the late 1960s by establishing regional branch campuses outside the Indianapolis metropolitan area (Figure 1). Half of the matriculating students are assigned to the main campus at Indianapolis, where they complete all four years of medical school. The other half are assigned to one of eight basic science campuses associated with local universities: Bloomington, Evansville, Fort Wayne, Gary, Muncie, South Bend, Terre Haute, and West Lafayette. After finishing their preclinical training, these students transfer to Indianapolis to complete their third and fourth years. Although other medical schools employ regional campuses for clinical training [12], IUSM is unusual in its reliance on regional campuses for basic science training, which offers a novel factor worthy of study. It has been generally assumed, though largely unsubstantiated, that IUSM students who are exposed to the training environments of the regional campuses will be predisposed to eventually return to those regions to practice, and that they will be more inclined to practice primary care medicine. Whether this approach to medical education truly influences students to pursue primary care careers in non-metropolitan areas has not been rigorously evaluated.

At a time when many of the nation's medical schools are proposing regional campuses as a way to increase the supply of physicians in their states [13], we thought it especially relevant to critically evaluate IUSM's four-decade experience in distributed medical education and assess its effectiveness in providing primary care practitioners to Indiana communities. To this end, we examined a ten-year cohort of IUSM graduates (1988–1997) and determined their medical specialties and practice locations as of 2003. Using multivariate logistic regression, we tested the hypothesis that training at regional basic science campuses influences IUSM students to pursue primary care careers near the regional campuses they attended. To the best of our knowledge, this is the first study to systematically analyze the novel factor of preclinical training site on career outcomes.

## Methods

### Study population

In this historical cohort study, we traced the career outcomes of 2,487 physicians who graduated from IUSM in the decade 1988–1997. Student information from school records was matched with data from the 2003 American Medical Association Physician Masterfile (Medical Marketing Service, Inc., Wood Dale, Illinois) to obtain the self-reported medical specialty and practice location of each graduate. When we initiated this study in 2004, the 2003 Masterfile was the most recent data available to match with student records. Assuming six years of post-graduate training thereby fixed 1997 as the upper limit of our study decade. We reasoned that by 2003, even the 1997 graduates would likely have completed residency training and settled into practice. In fact, we were able to find practice information in the Masterfile for virtually all of our graduates; only 26 of the 2,513 graduates from this decade were not accounted for in the Masterfile. According to the "type of practice" variable in the Masterfile, at least 92% of the study cohort self-reported to be active physicians with "direct patient contact." The research was granted approval by the University's Institutional Review Committee.

### Campus locations

The Indiana University statewide system of medical education consists of the main campus at Indianapolis and eight regional basic science campuses located at Bloomington, Evansville, Fort Wayne, Gary, Muncie, South Bend, Terre Haute, and West Lafayette (Figure 1). The nine cities containing IUSM campuses range in size from 29,000 (West Lafayette) to 786,000 (Indianapolis), and all of the cities other than Indianapolis have populations under 250,000. The racial composition of these communities is predominately white (66.1%–87.0%), with the exception of Gary, which is predominately black (84.0%). The racial composition of the state as a whole is 88.1% white and 9.0% black. The percentage of persons aged 65 and older in these communities ranges from 7.7% to 16.2%, with a mean of 12.3%. The percentage of college-educated persons ranges from 10.1% to 69.7%, with a mean of 28.3%. Additional socio-demographic details about Indiana can be found at: .

### Campus assignment

The assignment of students to a regional campus or Indianapolis is not random, but is based on a combination of student preference, availability of space, and the School's diversity needs (e.g., equitable gender distribution). When students are notified of their acceptance into medical school, they are asked to rank order their preferred campus assignment. The campus assignment process occurs after acceptance into medical school and has no bearing on the admission decision (e.g., a student cannot enhance his or her chances of admission by expressing a desire to attend a regional campus). Certain categories of accepted students automatically receive their first choice of campus assignment (e.g., early decision applicants), whereas others are not given the option of campus preference and are assigned strictly on the basis of available space (e.g., late admits taken from the alternate list). A student who owns a home near a campus or who is married to a spouse employed near a campus will be assigned to that campus if requested. Other special circumstances are occasionally honored when placing a student, but such cases are rare.

An analysis of three years of admission data (1995–1997) revealed that 69.8% of the newly matriculated students ranked Indianapolis as their first choice of campus assignment. The percentage of students who ranked a regional campus as their first choice ranged from 14.1% to 0.4%, depending on the campus. On average, any particular regional campus was ranked as a first choice by only 3.8% of the matriculating students. The reality is that no regional campus, with the exception of Bloomington, could fill its entering class with students who ranked that campus as their first choice assignment. By necessity, most students are assigned to a regional campus against their wishes and would have preferred Indianapolis. Those assigned to Indianapolis, on the other hand, are invariably there by choice and ranked it first on their list. Historically, 85% of the incoming students receive one of their top three campus choices. According to the 1995–1997 admissions data, in a typical entering class of 16–18 students at a regional campus (excluding Bloomington), only five or six students would have ranked that regional campus as their first choice assignment. This indicates that a minority of students self-select to attend regional campuses other than Bloomington, which tends to be a popular choice simply because it's the flagship campus of Indiana University, where many of our matriculating students attended college. Campus preference data were not available for the students in this study, but we have no reason to suspect their preferences would have been any different from those of the 1995–1997 matriculants.

At the time the study cohort entered IUSM, the School was admitting 280 students per year and apportioning them as follows: Indianapolis (138), Bloomington (28), Gary (18), Evansville (16), Fort Wayne (16), Muncie (16), South Bend (16), Terre Haute (16), and West Lafayette (16).

### Dependent variables

For each graduate in the dataset, we created two dichotomous outcome variables: one to indicate whether or not the graduate was practicing in a primary care specialty (medical specialty choice) and another to indicate whether or not the graduate was practicing in a particular geographical region associated with an IUSM campus (practice location choice). Those graduates who listed family medicine, general internal medicine, or general pediatrics as their practice specialty in the Masterfile were considered primary care physicians (coded as 1 in the dataset), whereas those in other specialties were considered non-primary care physicians (coded as 0 in the dataset).

To establish geographical boundaries, we divided the state into nine non-overlapping campus regions, one for each IUSM campus (Figure 1). Each campus region consisted of the county containing the IUSM campus and a surrounding cluster of contiguous counties. These campus regions were not defined arbitrarily, but rather represented each campus's recognized sphere of influence with regard to fund-raising, community support, and clinical affiliations. With the exception of the Indianapolis region, all of the campus regions contained sizable rural populations ranging from 20.0% to 69.3% of the total regional population. Depending on the region, the percentage of citizens living in Health Professional Shortage Areas (HPSAs) ranged from 7.8% to 23.2%, and the percentage living in Medically Underserved Areas (MUAs) ranged from 6.5% to 44.9%. In seven of the nine regions, the percentage of citizens living in MUAs exceeded 20%, and in two of the regions it exceeded 40%.

If a graduate's practice location fell within the boundaries of a given campus region, then that was coded as 1 in the dataset, otherwise 0. Each of the nine campus/practice regions was evaluated separately.

### Independent variables

Variables were selected for inclusion in the logistic regression models if chi-square tests revealed statistically significant associations with one or both of the dependent variables. Most of the independent variables selected for analysis have previously been shown to influence medical students' career choices [8,14].

For each graduate in the dataset, we created a series of dummy variables to indicate which of the nine IUSM campuses (campus regions) he/she attended. In the regression models for practice location choice, these variables were specifically coded to reflect the concordance between practice region and campus region. For example, if a graduate's practice location fell within the boundaries of the campus region he/she attended, then that was considered practicing in the vicinity of the IUSM campus. In other words, there was a concordance between practice region and campus region (coded as 1 in the dataset). Conversely, if a graduate's practice location fell outside the boundaries of the campus region he/she attended, then that was considered a discordance between practice region and campus region (coded as 0 in the dataset).

Other independent variables in the dataset included age at graduation, sex, race, and socioeconomic status (SES), which was based on the highest-income parent using a modification of the Hollingshead Index of Social Position [15]. Each graduate's self-reported hometown location and its urban influence code were recorded as separate variables. The urban influence code was based on the 1993 U.S. Department of Agriculture classification scale [16]. The year 1993 was chosen because it represented the approximate mid-point of the study decade. Each graduate's composite score on the Medical College Admission Test (MCAT) and his/her academic rank in medical school were converted to z-scores, and the constant 5 was added to each score to eliminate negative numbers. The academic rank was calculated as the equally-weighted average of three parts: the combined 1^st ^and 2^nd ^year grade point average, the score on U.S. Medical Licensing Exam (USMLE) Step 1, and the 3^rd ^year grade point average. This is the same academic rank that is currently reported in the Medical Student Performance Evaluation letters for graduating seniors. Each graduate's practice type was delineated as being either primary care or non-primary care. For those graduates who completed residency training in Indiana, we created variables to indicate the locations of their residency programs. All of the variables expressing geographic location (campus, practice, hometown, and residency) were based on zip codes. If the location of a graduate's hometown or residency program fell within the boundaries of his/her practice region, then that was coded as 1 in the dataset, otherwise 0. With the exception of age at graduation, MCAT score, and academic rank, all of the independent variables were dichotomous.

### Statistical analysis

Univariate analyses (t-test or chi-square) were performed to assess differences in the demographic and academic characteristics of students assigned to the main campus at Indianapolis versus students assigned to the regional campuses. Multivariate logistic regression was performed to assess the influence of regional campus attendance on students' choice of medical specialty and practice location, while simultaneously adjusting for several covariates thought to affect these outcomes. Each outcome (specialty choice and location choice) was evaluated separately.

To evaluate medical specialty choice, we included all graduates in the dataset, regardless of whether they were practicing in or out of state (N = 2,487). Academic rank was unavailable for 725 graduates (classes of 1988–1990). Eliminating the graduates with missing data elements and those with atypical campus assignments (e.g., repeating students, transfers, etc.) yielded 1,643 cases for analysis. Logistic regression models included the independent variables for campus region, age at graduation, sex, race, SES, hometown urban influence code, MCAT score, and academic rank. Two variants of the dependent variable were evaluated in separate regression models: whether or not a graduate was practicing a primary care specialty and, more specifically, whether or not a graduate was practicing family medicine.

To evaluate practice location choice, we excluded from the dataset 1,087 graduates who were practicing outside Indiana, because their practice locations were not germane to our hypothesis. Eliminating the graduates with missing data elements and those with atypical campus assignments yielded 1,294 in-state cases for analysis. Logistic regression models included the independent variables for campus region, age at graduation, sex, race, SES, hometown urban influence code, hometown location, residency location, and practice type. There were nine separate regression models, one for each IUSM campus (campus region). The likelihood of practicing in a given Indiana region–after attending the first two years of medical school in that region–was estimated separately for each campus.

Additional logistic regression models considered the regional campuses collectively, and evaluated whether attendance at any regional campus was more likely to result in (1) primary care specialty choice and (2) Indiana practice locations outside the Indianapolis metropolitan area.

The influence of regional campus attendance on students' choice of medical specialty and practice location was estimated by adjusted odds ratios (ORs) with 95% confidence intervals. All statistical analyses were performed using SPSS version 14.0 (Chicago, Illinois).

## Results

### Cohort demographics

Of the 2,487 physicians in the study cohort, 88.1% were white, 66.6% were male, 37.6% were primary care doctors, and 54.9% were practicing in Indiana. For the purposes of this study, primary care was defined to include family medicine, general internal medicine, and general pediatrics.

### Campus assignment

As shown in Table 1, the students who were assigned to a regional campus for their first two years of medical school differed slightly from those assigned to the main Indianapolis campus. Compared to Indianapolis students, those who attended a regional campus were somewhat more likely to be white, have parents with middle class occupations, and score marginally lower on the MCAT. However, any such differences between the two student populations were adjusted for in the regression analyses.

**Table 1:** Characteristics of Indiana University Medical Students Trained at a Regional Campus Versus Those Trained at the Main Indianapolis Campus, Graduating Classes of 1988–1997*

**Characteristic**	**Regional Campus** **(N = 1,211)**	**Indianapolis Campus** **(N = 1,200)**
Age at Graduation (mean, SD)	27.7, 3.1	27.7, 3.4
N	1,211	1,200
Sex (% female)	31.6%	33.8%
N	1,211	1,200
Race (% non-white)†	8.1%	11.2%
N	1,207	1,199
Socioeconomic Status (% middle & lower tier)†	42.5%	35.0%
N	1,193	1,182
Hometown Urban Influence Code (% non-metro)	19.2%	15.8%
N	1,206	1,187
MCAT Score (mean, SD)†‡	4.8, 0.95	5.0, 1.0
N	1,204	1,197
Academic Rank (mean, SD)§	5.0, 0.82	5.0, 0.92
N	853	833

*Of the 2,487 graduates in the study cohort, 76 were excluded because they had attended more than one campus during their preclinical years or because they had transferred into the 3^rd ^year from another school.

†Significantly different by two-tailed univariate test, P < 0.005.

‡Because MCAT scores have been scaled differently over the decade, and therefore not comparable across years, the students' scores were converted to z-scores and the constant 5 was added to provide a consistent scale.

§Each student's academic rank in medical school was computed as the equally-weighted average of three parts: combined 1^st ^and 2^nd ^year GPA, Step 1 score, and 3^rd ^year GPA. Rankings were converted to z-scores plus the constant 5. Academic rank was unavailable for 725 students (classes of 1988–1990).

### Medical specialty choice

**Figure 2:**
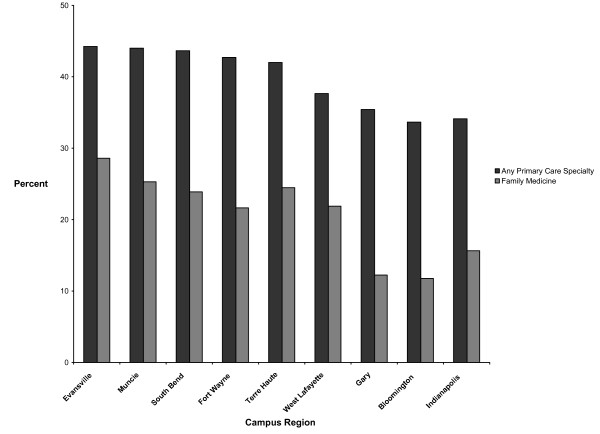
**Percentage of 1988 to 1997 Indiana University medical graduates trained at a regional campus or Indianapolis who, in 2003, were practicing any primary care specialty or family medicine in particular**.

Figure 2 shows the percentage of 1988 to 1997 graduates trained at a regional campus or Indianapolis who, in 2003, were practicing any primary care specialty or family medicine in particular. Of all the graduates who trained at the Evansville campus during the study decade, 44.2% subsequently entered primary care practice and 28.6% subsequently entered family practice. The Bloomington campus produced the lowest percentage of primary care practitioners (33.6%) and family practitioners (11.8%). All of the regional campuses except Bloomington produced a larger percentage of primary care practitioners than did Indianapolis. All of the regional campuses except Bloomington and Gary produced a larger percentage of family practitioners than did Indianapolis.

Compared to Indianapolis students, those who attended Evansville or Muncie for the first two years of medical school were 77% and 58%, respectively, more likely to enter primary care practice–controlling for age, sex, race, SES, hometown urban influence code, MCAT score, and academic rank (Additional file 1). Students who attended any regional campus, regardless of location, were 32% more likely to enter primary care practice.

Students who attended Evansville, South Bend, Muncie, or Terre Haute were significantly more likely (ORs = 2.43 to 1.74) to enter family practice, as compared to Indianapolis students (Additional file 1). Students who attended any regional campus, regardless of location, were 41% more likely to enter family practice.

### Practice location choice

**Figure 3:**
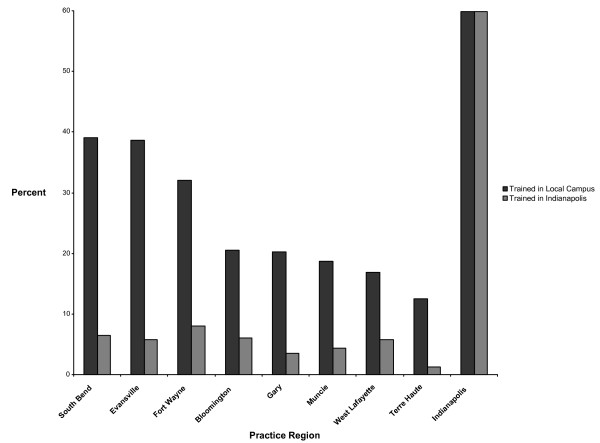
**Percentage of 1988 to 1997 Indiana University medical graduates trained at a regional campus or Indianapolis who, in 2003, were practicing in vicinity of the campus they attended**. Data do not include graduates who left Indiana to practice elsewhere.

Figure 3 shows the percentage of 1988 to 1997 graduates trained at a regional campus or Indianapolis who, in 2003, were practicing in the vicinity of the campus they attended. Each practice region associated with a regional campus had a higher proportion of locally-trained graduates than Indianapolis-trained graduates. For example, of all the graduates who trained at the South Bend campus during the study decade, 39.1% subsequently set up practice in the South Bend region. By contrast, only 6.4% of the Indianapolis-trained graduates set up practice in the South Bend region. Most (59.9%) of the Indianapolis-trained graduates established their practices in the Indianapolis region.

Compared to students from the other regional campuses, those who attended Terre Haute, South Bend, Bloomington, West Lafayette, or Evansville for the first two years of medical school were significantly more likely (ORs = 8.07 to 2.20) to return to those campus regions to practice–controlling for age, sex, race, SES, hometown urban influence code, hometown location, and practice type (Additional file 2). Students who attended any regional campus, regardless of location, were 34% more likely to establish a practice location outside the Indianapolis region.

Terre Haute, South Bend, and Evansville all have family medicine residency programs exclusively. When residency location was included as a covariate in the regression models, attendance at Terre Haute remained a statistically significant predictor of practice location choice, but not attendance at South Bend or Evansville (data not shown). The OR values for Bloomington and West Lafayette, which have no residency programs of any kind, were unchanged.

## Discussion

The results of this study support the hypothesis that training at regional basic science campuses influences IUSM students to pursue primary care careers near the regional campuses they attended. For the graduating classes of 1988–1997, attending a regional campus was a significant predictor of both medical specialty choice and practice location choice in logistic regression models that incorporated several covariates known to influence these career decisions. According to the regression models, four of the regional campuses were significantly more likely than Indianapolis to produce family practitioners, and five of the regional campuses were significantly more likely than the others to have former students practicing in the region. When analyzed collectively, attendance at any regional campus was a significant predictor of a primary care practice located outside the Indianapolis metropolitan area. These findings suggest that the regional campus environment during the first two years of medical school predisposes some students to pursue different career paths than those exposed only to academic medical centers. In other words, the preclinical training site can influence students' career choice independently of other variables.

Predicting career decisions in primary care medicine is a complex challenge involving numerous interacting variables. According to the Bland-Meurer Model of medical career decision-making, three principal factors influence specialty choice: (1) student characteristics, (2) medical school characteristics, and (3) students' perceptions of medical specialties [17]. Most studies have focused on the student characteristics that portend a primary care residency choice. Lawson and Hoban [14] reviewed six multivariate studies and found that students who go into primary care tend to be older than their classmates, female, belong to an underserved minority, have parents of lower socioeconomic status, have a rural hometown, lower MCAT scores, lower ratio of educational debt to expected income, and decided their specialty preference before medical school.

Where young physicians choose to establish their practices is likewise affected by the personal characteristics they bring as students. Having a rural or small town background seems especially influential. Laven and Wilkinson [8] identified 12 case-control or cohort studies that made quantitative comparisons between rural and urban doctors. They found that rural background was associated with rural practice in 10 of the 12 studies, and that students with a rural background were about twice as likely (OR = 2.0 to 2.5) to establish a rural practice compared to other students. In a previous study of IUSM graduates, Indiana physicians from non-metro hometowns were 4.7 times more likely to choose a non-metro practice location compared to their peers from metro hometowns, adjusting for age and sex [9]. The authors concluded that Indiana physicians from small hometowns have a strong preference to practice in regions similar to their hometowns if not actually near their hometowns.

By including several of these student characteristics as covariates in the regression models, we effectively controlled for their confounding influence on career choice to reveal an independent effect of regional campus attendance. As far as we are aware, no other study has examined the role of regional basic science campuses in this regard. Yet placed in their broader context, our findings are consistent with previous studies of medical students' career decisions. A variety of medical school programs designed to foster interest in primary care medicine have been shown to have a beneficial impact on the number of students entering primary care fields, often in medically underserved communities [10-12,18-20]. A key factor in the success of these programs appears to be primary care experiences during training, especially in community-based practice settings. Even experiences as early as the first year of medical school seem to positively impact residency choice [19]. However, Rabinowitz et al. [10] noted that students with rural backgrounds and early intentions to enter primary care were almost as likely to become rural primary care physicians as were students with similar characteristics but exposed to additional curricular experiences in primary care (e.g., an elective senior-year rural family medicine preceptorship). These authors concluded that a student's background and early career plans are the most important determinants of a career in primary care medicine, but special curricular experiences and other factors can enhance this outcome.

In the WWAMI program (acronym for Washington, Wyoming, Alaska, Montana, Idaho), students complete the first-year basic science coursework at their home state campus, and then move to the main Seattle campus for their second year. Students complete the 3^rd ^and 4^th ^year clinical rotations at sites of their choosing in the five-state region. Graduates of this program are reported to have higher rates of return to their home states for practice than the national average [21]. Although not directly comparable to the IUSM system, the WWAMI program does share certain features (e.g., preclinical training at regional campuses) and produces similar outcomes.

It is not intuitively obvious why attending a regional campus for the first two years of medical school would be conducive to a primary care career. Unlike schools with clinical branch campuses, where 3^rd ^and 4^th ^year students receive much of their clinical training from community physicians [12], the clinical education at IUSM's regional campuses is largely limited to the first- and second-year Introduction to Medicine courses. Nevertheless, according to the regression models, IUSM students who attended Evansville, South Bend, Muncie, or Terre Haute had a disproportionate propensity to become family physicians relative to their Indianapolis peers, adjusting for seven covariates known to affect specialty choice. What is it about these four regional campuses that could promote such an outcome? The formal curriculum is unlikely to be responsible because it is essentially the same at all sites. But each regional campus does have its own unique training environment, characterized in part by its network of connections with the local clinical community. This aspect is especially important because IUSM students at the regional campuses tend to have greater exposure to family physicians and other primary care providers than do their 1^st ^and 2^nd ^year counterparts at the Indianapolis campus, who tend to have greater exposure to specialists. Frequent interactions with these physician educators early in medical school may help to shape positive attitudes towards the primary care fields and family medicine in particular. Perhaps the four regional campuses in question have a greater involvement of family physicians in their educational programs than do the other campuses. Further studies will be needed to ascertain whether this possibility or other factors are responsible for the pronounced effect on specialty choice.

Equally intriguing is the pronounced effect of regional campus attendance on practice location choice. Our regression models predicted that IUSM students who attended Terre Haute, South Bend, Bloomington, West Lafayette, or Evansville were much more likely to return and practice in the surrounding area than were the students who attended other regional campuses, adjusting for seven influential covariates, including hometown location. The study cohort completed medical school in the decade 1988–1997, which means that graduates would have finished their specialty training and established their practices in the approximate period 1991–2003, assuming 3–6 years of postgraduate training. Because the information about medical specialty and practice location was drawn from the 2003 AMA Physician Masterfile, our findings should be viewed as a "snapshot" of the cohort as it existed in 2003. The state of affairs before or after this year is unknown. However, given the age of the cohort, it is reasonable to assume that most of the graduates were still in their first practice locations in 2003, and that relatively few had moved or changed their specialties.

Why would a student who attended a regional campus be drawn back to the area several years later to establish a practice? Proximity to the student's hometown cannot be the reason because hometown location was controlled for in the regression models. Other influences must be responsible. Relatively little is known about how new physicians decide initial practice locations, but in their survey of third-year family medicine residents, Costa et al. [22] found that spousal influence and favorable community characteristics were the most important determinants of location choice. We speculate that some of the IUSM students who attend regional campuses develop an affinity for these smaller communities and the family-friendly lifestyles they engender. As the students become familiar with the local hospitals, physicians, and patients, they come to appreciate what it might be like to practice in the community and envision similar opportunities for themselves. After graduation, these favorable impressions may influence their choice of practice location.

In some cases, a graduate's choice of a particular residency program may signal his or her intention to practice in a given community. For example, it is likely that several of the IUSM graduates who entered family medicine residency programs in South Bend and Evansville had previously decided to establish their practices in those regions. In such instances, residency location choice would be a proxy for practice location choice, which would confound the statistical relationship between independent and dependent variables in the regression models. This may explain why the South Bend and Evansville campuses were no longer significant predictors of practice location choice when residency location was included as a covariate. It is also possible that residency training in those two cities had a strong and independent effect on practice location choice, thus obscuring the effect of regional campus training.

### Study limitations

This was an observational study with no provision to randomly assign students to a campus. We therefore had to rely on multivariate regression techniques to adjust for any biases that may have resulted from the non-random assignment. No information was available about the students' campus preferences, so we could not adjust for this variable. In theory, some students may have self-selected to attend certain regional campuses based on their desire to eventually practice primary care medicine in those same regions. In practical terms, however, this seems unlikely to fully account for our findings because relatively few entering students self-select to attend any regional campus except Bloomington. Moreover, the fact that a student preferences a regional campus as his/her first choice assignment does not necessarily imply an early career decision. There are a variety of other reasons why a student might preference a particular regional campus, including its association with the student's undergraduate college, its unique learning environment and small class size (e.g., Problem-Based Learning and other non-traditional teaching modalities are offered at some campuses), and economic incentives (e.g., some of the regional campuses offer scholarship inducements to attend). Evidence that self-selection is unlikely to explain our results can be seen from the outcomes at specific campuses (Additional files 1 and 2). For example, the Terre Haute campus attracts the fewest students of any regional campus (an average of only one student per year) and yet it produces a disproportionate number of graduates who return to practice family medicine in the area. Conversely, the Gary campus attracts a third of its class (an average of seven students per year) and yet it does not have a significant impact on the supply of local primary care providers. Despite these observations, we cannot rule out the possibility that self-selection may have confounded our findings to some degree. No information was available about the students' debt load or their specialty preferences prior to medical school, both of which have been implicated as factors in career decision-making [10,23].

As mentioned previously, the students' clinical experiences at certain regional campuses, and the particular kinds of interactions they have with local physicians, may account for much of their predilection toward primary care careers. In our judgment, fully understanding how the clinical experiences at regional campuses differ from those at Indianapolis is key to explaining the campus effect.

Our study cohort completed medical school approximately 10–20 years ago, at a time when the economic forces and practice conditions affecting new physicians were somewhat different than they are now. In the intervening years, the composition of the IUSM student body has changed as well, with greater ethnic diversity and gender balance. How the career decisions of today's medical students are being shaped by their regional campus experiences remains to be determined. Other outcomes may manifest in a later cohort of graduates. Finally, IUSM is a large, midwestern medical school with educational traditions and demographic characteristics that may differ substantially from other schools in other locales. Our findings may not generalize to other student populations.

## Conclusion

Regional campus attendance–even for the preclinical years–appears to have lasting influences on the career decisions of IUSM graduates. For reasons yet to be fully explicated, attending a regional basic science campus increases the likelihood of practicing primary care medicine, especially family medicine, outside the major metropolitan areas. Many state legislatures and medical schools are currently grappling with the task of expanding the physician workforce in anticipation of future health care demands. Our findings provide empirical evidence suggesting that regional campuses might be an effective way to increase the number of primary care physicians in communities where they are most needed.

## Competing interests

The authors declare that they have no competing interests.

## Authors' contributions

JJB conceived and designed the study and wrote the draft manuscript. CAM and MEW performed the statistical analyses. DDW and GWW constructed the database of study variables. MTJ provided data about students' regional campus choices. JSW created the map of IUSM campuses. TWZ contributed to the study design and interpretation of data and revised the draft manuscript. All authors read and approved the final manuscript.

## About the authors

JJB is an Associate Dean in the Office of Medical Student Affairs, Indiana University School of Medicine, Indianapolis. CAM is a data manager/protocol monitor for The EMMES Corporation, Rockville, MD. MEW is a syndromic surveillance epidemiologist for the Indiana State Department of Health, Indianapolis. DWD is the Director of Student Records in the Office of Medical Student Affairs, Indiana University School of Medicine, Indianapolis. MTJ is an Associate Professor in the Department of Microbiology and Immunology, Indiana University School of Medicine, Terre Haute. GWW is a Lecturer in the Department of Anatomy and Cell Biology, Indiana University School of Medicine, Evansville. JSW is an Associate Professor and Chair in the Department of Geography, Indiana University-Purdue University, Indianapolis. TWZ is a Professor and Associate Director in the Bowen Research Center, Indiana University School of Medicine, Indianapolis.

## Pre-publication history

The pre-publication history for this paper can be accessed here:



## Supplementary Material

Additional File 1 **Table 2**. Influence of regional campus training on medical specialty choice of Indiana University medical students. Click here for file

Additional File 2 **Table 3**. Influence of regional campus training on practice location choice of Indiana University medical students. Click here for file
