# Supplementary material for: The influence of regional basic science campuses on medical students' choice of specialty and practice location: a historical cohort study
Source: BMC Med Educ. 2009 Jun 6;9:29. doi: 10.1186/1472-6920-9-29 (PMC2700105; doi:10.1186/1472-6920-9-29)
Supplement: Additional File 1 — Table 2. Influence of regional campus training on medical specialty choice of Indiana University medical students. [file 1472-6920-9-29-S1.doc]

Table 2: Influence of Regional Campus Training on Medical Specialty Choice of Indiana University Medical Students, Graduating Classes of 1988–1997 (N = 2,487)

| **Dependent Variable*** | **Independent Variable†** | **Adjusted Odds Ratio‡** | **95% Confidence Interval** | **P Value** |
| --- | --- | --- | --- | --- |
| Medical Specialty Choice | Campus Region |  | | |
| Any primary care specialty | Evansville | 1.77 | 1.15–2.70 | 0.009 |
|  | Muncie | 1.58 | 1.03–2.41 | 0.035 |
|  | Terre Haute | 1.46 | 0.94–2.26 | 0.092 |
|  | South Bend | 1.45 | 0.93–2.26 | 0.100 |
|  | Fort Wayne | 1.42 | 0.93–2.17 | 0.107 |
|  | West Lafayette | 1.21 | 0.78–1.87 | 0.401 |
|  | Gary | 1.04 | 0.67–1.61 | 0.858 |
|  | Bloomington | 1.01 | 0.69–1.47 | 0.961 |
|  | Any Regional Campus | 1.32 | 1.08–1.62 | 0.008 |
|  | | | | |
| Family medicine | Evansville | 2.43 | 1.53–3.88 | 0.001 |
|  | South Bend | 1.91 | 1.15–3.17 | 0.012 |
|  | Muncie | 1.78 | 1.10–2.87 | 0.018 |
|  | Terre Haute | 1.74 | 1.05–2.87 | 0.031 |
|  | Fort Wayne | 1.42 | 0.86–2.37 | 0.174 |
|  | West Lafayette | 1.42 | 0.85–2.40 | 0.184 |
|  | Gary | 0.75 | 0.41–1.37 | 0.354 |
|  | Bloomington | 0.64 | 0.36–1.11 | 0.111 |
|  | Any Regional Campus | 1.41 | 1.09–1.81 | 0.009 |
|  | | | | |

*Multivariate logistic regression involving 1,643 graduates practicing inside or outside Indiana. Excluded from the analysis were 844 graduates with missing data elements or atypical campus assignments (e.g., repeating students and transfers from other schools).

†Covariates included age at graduation, sex, race, socioeconomic status, hometown urban influence code, MCAT score, and academic rank in medical school.

‡Relative to the Indianapolis campus.
