# Supplementary material for: The influence of regional basic science campuses on medical students' choice of specialty and practice location: a historical cohort study
Source: BMC Med Educ. 2009 Jun 6;9:29. doi: 10.1186/1472-6920-9-29 (PMC2700105; doi:10.1186/1472-6920-9-29)
Supplement: Additional File 2 — Table 3. Influence of regional campus training on practice location choice of Indiana University medical students. [file 1472-6920-9-29-S2.doc]

Table 3: Influence of Regional Campus Training on Practice Location Choice of Indiana University Medical Students, Graduating Classes of 1988–1997 (N = 2,487)

| **Dependent Variable*** | **Independent Variable†** | **Adjusted Odds Ratio‡** | **95% Confidence Interval** | **P Value** |
| --- | --- | --- | --- | --- |
| Practice Location Choice | Campus Region |  | | |
| Practice inside campus region | Terre Haute | 8.07 | 2.63–24.73 | 0.001 |
|  | South Bend | 4.47 | 2.33–8.56 | 0.001 |
|  | Bloomington | 3.35 | 1.89–5.95 | 0.001 |
|  | West Lafayette | 2.51 | 1.24–5.11 | 0.011 |
|  | Evansville | 2.20 | 1.15–4.22 | 0.017 |
|  | Gary | 2.01 | 0.88–4.63 | 0.099 |
|  | Muncie | 1.90 | 0.90–4.03 | 0.093 |
|  | Fort Wayne | 1.59 | 0.85–2.96 | 0.148 |
|  | | | | |
| Practice outside Indianapolis | Any Regional Campus | 1.34§ | 1.06–1.71 | 0.015 |
|  | | | | |

*Multivariate logistic regression involving 1,294 graduates with Indiana practice locations. Excluded from the analysis were 1,087 graduates with out-of-state practice locations and 106 graduates with missing data elements or atypical campus assignments (e.g., repeating students and transfers from other schools).

†Covariates included age at graduation, sex, race, socioeconomic status, hometown urban influence code, hometown location, and practice type.

‡Relative to any other campus.

§Relative to the Indianapolis campus.
